# Machine Learning (ML) based-method applied in recurrent pregnancy loss (RPL) patients diagnostic work-up: a potential innovation in common clinical practice

**DOI:** 10.1038/s41598-020-64512-4

**Published:** 2020-05-14

**Authors:** V. Bruno, M. D’Orazio, C. Ticconi, P. Abundo, S. Riccio, E. Martinelli, N. Rosato, E. Piccione, E. Zupi, A. Pietropolli

**Affiliations:** 1 Academic Department of Biomedicine and Prevention, University of Rome Tor Vergata, and Clinical Department of Surgical Sciences, Section of Gynecology, Tor Vergata University Hospital, Viale Oxford 81 - 00133 Rome, Italy; 2 Academic Department of Surgical Sciences, Section of Gynecology, Tor Vergata University Hospital, Viale Oxford 81 - 00133 Rome, Italy; 3 Medical Engineering Service and General Direction, Tor Vergata University Hospital, Viale Oxford 81 - 00133 Rome, Italy; 4 Department of Electronic Engineering, University of Rome Tor Vergata, Via del Politecnico 1 - 00133 Rome, Italy; 5 Academic Department of Experimental Medicine and Surgery, University of Rome Tor Vergata, and Medical Engineering Service and General Direction, Tor Vergata University Hospital, Viale Oxford 81 - 00133 Rome, Italy; 6 Department of Molecular Medicine and Development, University of Siena, University Hospital “S.Maria alle Scotte” Viale Mario Bracci, 53100 Siena, Italy

**Keywords:** Reproductive disorders, Translational research

## Abstract

RPL is a very debated condition, in which many issues concerning definition, etiological factors to investigate or therapies to apply are still controversial. ML could help clinicians to reach an objectiveness in RPL classification and access to care. Our aim was to stratify RPL patients in different risk classes by applying an ML algorithm, through a diagnostic work-up to validate it for the appropriate prognosis and potential therapeutic approach. 734 patients were enrolled and divided into 4 risk classes, according to the numbers of miscarriages. ML method, called Support Vector Machine (SVM), was used to analyze data. Using the whole set of 43 features and the set of the most informative 18 features we obtained comparable results: respectively 81.86 ± 0.35% and 81.71 ± 0.37% Unbalanced Accuracy. Applying the same method, introducing the only features recommended by ESHRE, a correct classification was obtained only in 58.52 ± 0.58%. ML approach could provide a Support Decision System tool to stratify RPL patients and address them objectively to the proper clinical management.

## Introduction

Recurrent pregnancy loss (RPL) is a very debated field: the absence of fully shared guidelines arises controversial issues in the clinical management of these patients, confusion about diagnostic work-up to be performed and potential therapies to be applied. This confusion is attributable to the impossibility of reaching an evidence-based-medicine, since the disparity, even on the definition of the pathology, makes the approach to the problem more complicated^1^. Furthermore, in the scientific community, no full agreement has been achieved on crucial factors, such as number of miscarriages to be considered for the definition (two versus three); consecutivity of miscarriage; inclusion in the definition of RPL of: “non-visualized pregnancy losses”, biochemical abortions, intrauterine clinical abortions, pregnancies of unknown location (PULs), ectopic or molar pregnancies.

The most recent international guidelines of the ESHRE (European Society of Human Reproduction and Embriology) define RPL as the loss of two or more pregnancies before the 24th week of gestation. With this settlement, the concept of consecutivity lapses and “non-visual” pregnancies, such as biochemical or PULs abortions, are also included in the definition, because of their weight in estimating the prognosis for unexplained recurrent pregnancy loss (uRPL) clinical cases.

In these latter guidelines, the clinical evaluation of the RPL couple is recommended whenever the second miscarriage occurs, since the incidence of positive results in the RPL diagnostic screening is similar in patients with two or more miscarriage compared to patients with three or more^2,3^. This decision already poses a change in the analysis of the problem within the scientific literature, as the previous ESHRE or RCOG (Royal College of Obstetrics and Gynecology) guidelines^4,5^ used higher thresholds and more restrictive criteria that excluded a large number of patients. Indeed, the prevalence of women affected by RPL ranges from 1% to 5%, according to which of the different definition for RPL is used^2^.

Owing to the above mentioned problems and pitfalls, it is reasonable to try to carry out a screening that allows to investigate the different critical RPL aspects in the best possible way, in order to classify patients in risk classes, taking into account the etiological factors in an interdisciplinary perspective.

To design a common shared diagnostic algorithm, it will be useful to define proven and probable risk and etiological factors for RPL and how scientific literature classifies them in relation to the disease onset.

Many risk factors, such as are medical and family history, age, stress, lifestyle, smoke, obesity, chronic endometritis and abnormal decidualization (both of them not recommended investigations by ESHRE guidelines 2017), may facilitate the onset of the disease: their absence does not exclude the disease, but their co-presence significantly increases the risk of the disease^6^. Nevertheless, there is not a clear and full scientific agreement between research groups in the field about the weight that some of these risk factors have in the etiopathogenesis of RPL.

Infections, chromosomal abnormalities, endocrinological and metabolic diseases, autoimmune diseases and immunological dysregulation, selected thrombophilia, uterine anatomical abnormalities are known etiological factor for RPL, but more than half of couples affected by RPL have no evident and direct cause for pregnancy failure, therefore it is not possible to proceed with an appropriate therapy, rather they are offered psychological support and lifestyle suggestions^2^.

This aspect highlights the possibility of representing RPL through a theoretical threshold model, i.e. the association of minor factors that may exceed a threshold value can cause the disease, even if taken individually they would not be relevant^6^.

This representation is reasonable, since RPL is a multifactorial disease and the analysis of patient results must be thought of in an overview and not as completely unplugged examinations.

The stratification in risk classes of patients affected by RPL would be aimed at: identifying RPL causes that occur most frequently, in order to deepen the several research lines in the field; ensuring a proper communication between clinicians and patients, highlighting any chances of success of pregnancy in terms of personalized prognosis; identifying a schematic and objective diagnostic and personalized therapeutic approach among the different available ones, on the basis of the results of the obtained classification; helping the communication of that particular therapy for potential contraindications and benefits.

This type of approach to the problem is in no way intended to replace the work of clinicians, but rather to provide a tool of Support Decision System, through which they can direct patients in different paths of access to care.

## Results

### Clinical characteristics of studied population

The population enrolled in the study represents an excellent photograph of the prevalence of each factor investigated in the diagnostic work-up performed. Number of abortions and age of patients are shown in Table 1. Regarding age, it is clear that RPL has been found more frequently going forward with the maternal age. The correlation that emerges between the number of abortions and the maternal age is summarized in Table 2.

**Table 1:** Clinical characteristics of studied population.

Categories		n° patients	%
**Age**	18–27	67	9.13
	28–32	149	20.30
	33–37	250	34.06
	38–42	209	28.47
	≥43	45	6.13
	NaN	14	1.91
**N° of abortions**	0	19	2.59
	1	79	10.76
	2	314	42.78
	3	194	26.43
	4	81	11.04
	≥5	47	6.40
**BMI**	<16.5	2	0.27
	16.5–18.49	27	3.69
	18.5–24.99	368	50.13
	25–30	132	17.99
	>30	62	8.44
	NaN	143	19.48
**Anatomical uterine abnormalities**	Bicorporeal uterus- class U3	3	0.41
	Septate uterus- class U2	53	7.22
	Hemi uterus - class U4	4	0.54
	Endometriosis	17	2.31
	Uterine Fibroids	112	15.26
	Intrauterine Synechiae	19	2.59
	Endometrial polyps	45	6.13
	Cervical-isthmic incontinence	5	0.68
**Coagulation abnormalities**	APCR	215	29.29
	Protein C	58	7.9
	Protein S	108	14.7
	AT III	35	4.77
	Factor V Leiden		
	• Eterozygosis	22	3
	• omozygosis	13	1.77
	Factor II		
	• Eterozygosis	29	3.95
	• omozygosis	3	0.41
	MTHFR + PAI-I		
	Score		
	• 1	101	13.76
	• 2	191	26.02
	• 3	129	17.57
	• 4	46	6.27
	• 5	1	0.14
	omocysteinemia	61	8.31
**Others**	Diabetes	30	4.09
	PCOS	28	3.81
	Dysthiroidisms	148	20.16
	Smoking	128	17.44
	Maternal chromosomal abnormalities	14	1.91
	Paternal chromosomal abnormalities	7	0.95
	Infections	102	13.9
**APS**	LAC, ACI, Ab anti-β2 GP1		
	Score:		
	• I Ab	49	6.67
	• 2 Abs	6	0.82
	• 3 Abs	1	0.14
	Ab anti-annexin V	19	2.59
**Abs anti-thyroid**	Ab anti-TPO, Ab anti-Tg		
	Score:		
	• I Ab	84	11.44
	• 2 Abs	24	3.27
	• NaN	10	1.36
**Celiac disease**	Ab anti-endomysium, Ab anti-transglutaminase, Ab anti-gliadin		
	Score:		
	• I Ab	7	0.95
	• 2 Abs	2	0.27
	• 3 Abs	4	0.54
**ANA**	Score:		
	• Negative	285	38.83
	• 1:80	148	20.16
	• 1:160	50	6.82
	• 1:320	10	1.36
	• 1:640	10	1.36
	• 1:>640	2	0.27
	• NaN	229	31.2
**Systemic autoimmunity**	ENA, AMA, ASMA		
	Score:		
	• 1 Ab	66	8.99
	• 2 Abs	10	1.36

For each clinical and etiological feature the incidence in our population is described (number and and percentage of patients). For coagulation abnormalities the incidence, in terms of numbers of patients with values which are outside the physiological ranges, is shown. In MTHFR + PAI-1 feature, a score which is the result of each single exam score (0 = negative for mutation, 1 = mutation in eterozygosis, 2 = mutation in omozygosis). Regarding APS, Abs anti-thyroid, Celiac disease, and systemic autoimmunity, the incidence for the presence in a single patient of only 1, or 2 or 3 antibodies of that one included in each category, is indicated.

**Table 2:** Number of pregnancy losses and maternal age.

Number of pregnancy losses	0	1	2	3	4	≥5
18–27 (age)	2	14	31	12	6	2
28–32 (age)	8	15	71	33	16	6
33–37 (age)	4	30	105	67	27	17
38–42 (age)	1	14	90	63	26	15
≥43	0	4	15	17	3	6

There is a greater prevalence in class 2 abortions with a peak in patients aged between 33–37 years. The classes with a high number of abortions are few in number, as patients often become resigned, leading to the abandonment of hospital facilities.

The body mass index data shows a high frequency of patients of normal weight. The clear difference between underweight and overweight patients, highlights the potential link between unbalanced BMI and RPL, especially for obese patients (Table 1).

For uterine abnormalities, the percentage of patients with this type of problem was very unbalanced compared to the general population, highlighting the correlation between RPL and uterine abnormalities (Table 1). For thrombophilic and immunological causes, as well as for all other risk factors, data are shown in Table 1.

### **Results for SVM method application**

Results are presented through a confusion matrix, that evaluates the correct classification of the algorithm, and a patient’s score.

In the two-class problem (healthy and sick), using all the available features, we obtained a 90.24 ± 0.36% balanced accuracy in Leave 200 Out Cross Validation (Table 3). Using only the selected 18 features we obtained 93.85 ± 0.34% balanced accuracy (Table 4).

**Table 3:** Two-classes analysis overall results. Model Trained on all the available features selected in leave 200 out cross validation over 1000 iterations: Confusion matrix.

2-classes analysis		Predicted class	
		Healthy	Affected (RPL)
Real class	*Healthy*	83.71 ± 0.72%	19.29 ± 0.72%
	*Affected (RPL)*	3.23 ± 0.16%	96.76 ± 0.16%

ACCub (unbalanced accuracy) = 93.51 ± 0.20%;

ACCb (balanced accuracy) =90.24 ± 0.36%.

**Table 4:** Two-classes analysis overall results. Model Trained on the 18 features selected in leave 200 out cross validation over 1000 iterations: Confusion matrix.

2-classes analysis		Predicted class	
		*Healthy*	*Affected (RPL)*
Real class	*Healthy*	88.98 ± 0.66%	11.01 ± 0.66%
	*Affected (RPL)*	1.29 ± 0.13%	98.71 ± 0.13%

ACCub (unbalanced accuracy) = 96.28 ± 0.19%;

ACCb (balanced accuracy) = 93.85 ± 0.34%.

In the four class Problem using all the available features we achieved 81.83 ± 0.35% Balanced Accuracy (confusion matrix shown in Table 5). We obtained 8.54 ± 0.63% overestimated patients and 9.59 ± 0.56% underestimated patients.

**Table 5:** Four-classes analysis overall results. Model Trained on the all the available features selected in leave 200 out cross validation over 1000 iterations: Confusion matrix.

		Predicted class			
		0 abortion	1 abortion	2–3 abortions	≥4 abortions
Real class	0 abortion	91.46 ± 0.33%	8.05 ± 0.21%	0.47 ± 0.25%	0.02 ± 0.06%
	1 abortion	0.93 ± 0.23%	89.39 ± 0.95%	7.59 ± 0.83%	2.08 ± 0.45%
	2–3 abortions	3.26 ± 0.22%	11.82 ± 0.50%	68.86 ± 0.76%	16.06 ± 0.69%
	≥4 abortions	2.55 ± 0.07%	8.67 ± 0.36%	11.17 ± 0.85%	77.62 ± 0.91%

ACCub (unbalanced accuracy) = 81.86 ± 0.35%;

ACCb (balanced accuracy) = 81.83 ± 0.35%.

Using only the subset of 18 features selected we obtained 81.55 ± 0.37% Balanced Accuracy (confusion matrix shown in Table 6). We obtained 7.88 ± 0.60% overestimated patients and 10.41 ± 0.61% underestimated patients.

**Table 6:** Four-classes analysis. Model Trained on the 18 features selected in leave 200 out cross validation over 1000 iterations: Confusion matrix.

		Predicted class			
		0 abortion	1 abortion	2–3 abortions	≥4 abortions
Real class	0 abortion	91.28 ± 0.34%	8.06 ± 0.21%	0.66 ± 0.27%	0.003 ± 0.02%
	1 abortion	0.91 ± 0.24%	88.97 ± 0.95%	8.15 ± 0.81%	1.96 ± 0.41%
	2–3 abortions	4.16 ± 0.26%	12.98 ± 0.45%	70.12 ± 0.72%	12.75 ± 0.63%
	≥4 abortions	2.63 ± 0.17%	9.94 ± 0.41%	11.09 ± 0.89%	76.35 ± 0.94%

ACCub (unbalanced accuracy) = 81.71 ± 0.37%;

ACCb (balanced accuracy) = 81.55 ± 0.37%.

The four-class problem offered more stratified results but a greater error probability with respect to the two-class problem.

The SVM algorithm returns also the probability of each patient of belonging to each class. The higher the probability the most likely the patient is to belong to that specific class.

### Results from procedures comparison: ESHRE guidelines versus our local recommendations

The comparison between the two different diagnostic work-ups is summarized in Table 7.

**Table 7:** Comparison between the two different diagnostic work-up: ESHRE guidelines versus our RPL Unit recommendations.

ESHRE guidelines	Our diagnostic work-up
**Medical and family history to tailor RPL diagnostic investigations**	Medical, obstetric and family anamnesis & Clinical characteristics (e.g. maternal age, BMI) and lifestyle
**Number of previous pregnancy losses and maternal age to establish prognosis**	Gynecologic clinical examination
**Parental karyotype not routinely recommended**	High resolution maternal and paternal karyotypes
**Screening for hereditary thrombophilia not recommended, except for women with additional risk factors for thrombophilia**	Factor V Leiden and factor II mutations, MTHFR A 1298 C and C 677 T polymorphisms, PAI-I mutation, APCR, protein C and S, ATIII, omocysteinemia
**Lupus anticoagulant (LAC) and anti-cardiolipin Abs (IgM, IgG) recommended**	Lupus anticoagulant (LAC), anti-cardiolipin Abs (IgM, IgG, IgA)
**Anti-β**_**2**_ **glycoprotein I Abs can be considered**	Anti-β2 glycoprotein I and anti-annexin V Abs
**ANA testing only for explanatory purposes**	ANA
**TSH and anti-TPO Abs recommended**	TSH assay, anti-TPO and anti-Tg Abs
**T4 testing as a follow-up for women with abnormal TSH and anti-TPO Abs**	FT3 and FT4 assays
**Assessment of uterine anatomy, preferably by a transvaginal 3D ultrasound**	3D transvaginal ultrasound
	Diagnostic hysteroscopy ± endometrial biopsy
	Vaginal-cervical swabs
	Oral glucose tolerance test and glycated haemoglobolin
	Anti-gliadin, anti-transglutaminase and anti-endomysium Abs
	Systemic autoimmunity: ASMA, ENA, anti-dsDNA Abs, AMA

Analyzing our problem with the only features included in the diagnostic work-up of the guidelines we obtained 58.51 ± 0.58% balanced accuracy (confusion matrix shown in Table 8), with 20.13 ± 1.56% overestimated patients and 21.35 ± 1.00% underestimated patients.

**Table 8:** Four-classes analysis overall results: confusion matrix from ESHRE guidelines diagnostic work-up.

		Predicted class			
		1 st	2 nd	3 th	4th
Real Class	*1 st*	76.20 ± 1.04%	13.32 ± 0.70%	3.89 ± 0.56%	6.59 ± 0.82%
	*2 nd*	15.92 ± 0.83%	52.19 ± 1.29%	10.40 ± 0.92%	20.48 ± 1.10%
	*3 th*	11.00 ± 0.37%	18.66 ± 0.61%	44.43 ± 2.17%	25.91 ± 2.16%
	*4 th*	6.74 ± 0.38%	13.53 ± 0.56%	19.50 ± 1.25%	60.22 ± 1.29%

ACCub (unbalanced accuracy) = 58.52 ± 0.58%;

ACCb (balanced accuracy) = 58.51 ± 0.58%.

These results demonstrate that the features recommended by ESHRE guidelines do not offer sufficient information to distinguish classes especially for class 2 and 3, in which there is the theoretical boundary between healthy and sick patients: acceptable results are obtained only for the class 0 abortions and the class ≥4 abortions, therefore, in the point of greatest interest (the “grey zone” where the boundary between “healthy” and “sick” has been established) the classification was not very efficient.

This type of analysis highlights a fairly important aspect: in order to find answers to past obstetric failures and future pregnancies, patients could undertake a more thorough diagnostic screening.

## Discussion and future perspectives

### Potential application of the obtained algorithm in the clinical practice

#### How to use the screening result

The main goal of this study was to build up a machine learning algorithm that stratifies patients into classes of risk, a new concept in this research field, starting from analyzing a dataset of RPL patients through their diagnostic work-up results and comparing them with the indications of ESHRE guidelines.

First, all the 43 features came out from our RPL diagnostic work-up results were used in order to let the algorithm predict patient’s risk class. Next step would have been to reduce the number of features needed for the prediction of the risk class, to use this prognostic model as a potential screening tool, more expendable in clinical practice in terms of cost/benefit ratio. This could have been achieved by verifying, through ML application, those features which weight more in correctly discriminating against one risk class from another. Then, by selecting only 18 features, as described in methods section, the obtained results were comparable.

In order to stratify the patients into risk classes, we tackled the problem in two and four classes. In both cases, studies offer excellent results and benefit.

In the two-class investigation, a correct classification was achieved: i.e. the class predicted by the classifier coincided with the actual class of the patient in 96.28 ± 0.19% (unbalanced accuracy, see Table 7) of the cases and offers the advantage of having a score function with a numerical result that represents the patient’s condition. In the four classes investigation, the percentage of correct classification offered by the algorithm using the subset of 18 features is 81.71 ± 0.37%, obtaining a single patient screening result as a percentage of belonging to the different risk classes.

For the screening of any new patients, an interface has been created which takes the features as input and offers as output a histogram with the percentage of belonging to the different classes of risk.

Depending on the result of the algorithm it would be possible to build up a specific clinical management for each RPL couple. In order to apply this method, it is necessary to understand if the patient has been correctly classified, that is, if the real class coincides with the predicted class. If the patient is not correctly classified, we have two cases: underestimated or overestimated risk class.

Underestimated means a patient who belongs to a higher risk class than the class predicted by the algorithm. A similar situation may arise when the real cause of the abortion events has not been discovered. Patients in this situation must undergo further diagnostic tests, otherwise in agreement with the clinician, participate in research trials on uRPL, since none of the causes known from the literature is attributable for their RPL history.

If, on the other hand, a patient belongs to a lower risk class than that predicted by the algorithm and has been overestimated, this means that it could be possible to identify a cause of past abortion events and, in agreement with the clinician, should perform further investigations or clinical procedures to solve these problems before trying a new pregnancy.

This procedure could also ensure a more realistic prognosis for further abortion events prediction and could guide the clinical management in further RPL patients pregnancies.

The correctness of such an analysis is strongly influenced by the number of patients used in the training phase, since the more the training set is numerous, the more the risk classes will have well-defined outlines. To have more patients enrolled in the study is a minimum requirement to ensure more reliable screening.

Furthermore, as a last step, we focus on the analysis of the potential application of the algorithm in the hospital procedure, by comparing our current work-up to that one recommended by the most recent guidelines.

Applying the algorithm to the two diagnostic work-ups analyzed, the differences highlighted are significant. The correct classification guaranteed by the selected features is 81.55 ± 0.37% (balanced accuracy see Table 6), while, with only the recommended diagnostic exams, the balanced accuracy drops dramatically to 58.51 ± 0.58% (see Table 8). This shows that a correct prognosis unavoidably entails higher costs but can guarantee a therapeutic approach and communication between the actors involved potentially more appropriate to the conditions of every single patient.

The work carried out provides a Support Decision System tool (currently completely absent in this field) in hospital practices.

In details, analyzing data with only the features included in the ESHRE guidelines diagnostic work-up, it turned out that the classification rate of each class was 76.20 ± 1.04%, 52.19 ± 1.29%, 44.43 ± 2.17% and 60.22 ± 1.29%, for the first, second, third and fourth class respectively (see Table 8) with an overestimated patient percentages of 20.13 ± 1.56% and an underestimated patient percentages of 21.35 ± 1.00%.

Moreover, the classification model built using only the features suggested by the guidelines offers almost acceptable results only for class 0 abortions and class ≥4 abortions, therefore, at the point of greatest interest (the “grey zone” where the boundary between “healthy” and “sick” has been established) the classification is not very efficient.

The application of the algorithm in predicting the risk class of the patients, could help the clinicians to decide a potential therapeutic approach to be assigned to each single patient as a personalized and targeted therapy: this can be considered as a Support Decision System method capable of objectively directing patients into post-screening pathways, ensuring uniformity of management for the different clinical cases.

The proposed method cannot and must not be considered flawless; like any innovative prototype, it is subject to further improvement. Therefore, the version of the algorithm provided in this paper, and thus used in the analysis, cannot be considered obviously the definitive one. Increasing the size of the dataset used we will able to reduce the impact of bootstrap on the performance and to add statistical relevance to the obtained results.

### Conclusions and future developments

In the near future, the potential objective could be a similar application to one applied in this study, designed to address and untangle other multifactorial issues. Among the desirable improvements, to subtract the current risk classes according to different “types of etiology” would be interesting, so that the treatment of care is assigned automatically and objectively. The idea behind a possible outcome would be developed through the creation of “typical patients”, with a group of “switched on” features on the highest risk class verifying whether the response of the algorithm coincides with what is the current clinical practice. The goal of classifying patients into risk classes and objective targeting them in therapeutic pathways according to the features can be considered widely achieved thanks to machine learning. The next step for this work to monitor the changes in patients’ risk class, according to the different therapeutic approaches applied. It would be also interesting to investigate features fluctuations throughout all pregnancy to have a predictive tool personalized for each pregnancy. As the last point, to outreach, the project to cover a wider dataset of patients and /or to compare different “national RPL recommendations” from different countries would be desirable.

To sum up, this work represents only a possible way to make this field as much as objectifiable as possible, not “The Way”. This attempt could be the first step to further investigations needed, especially in terms of international multicenter collaborations. We hope it could be a starting point to go further for patient’s care sake.

## Material and Methods

**Figure 1:**
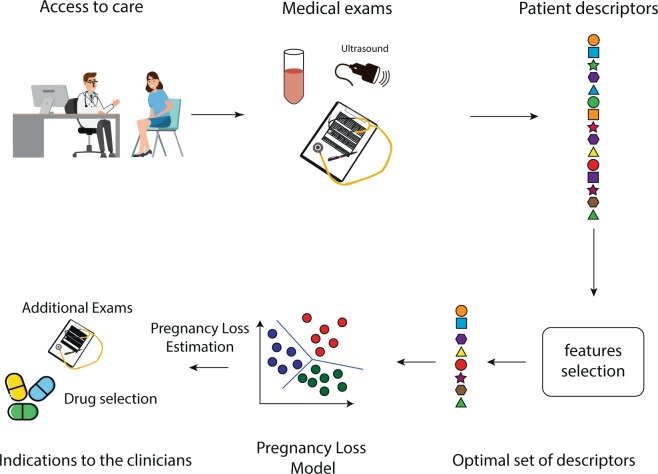
Method steps flow chart.

A flow chart of the whole method steps is represented in Fig. 1.

### Subjects

To carry out a stratification of the level of risk for each RPL patient, data from medical records of 734 non-pregnant women referred to the Section of Gynecology, Department of Surgical Sciences of Tor Vergata University Hospital were used. The enrolled women were divided into “healthy population” (patients who have undergone at most one abortion and at least two pregnancies at terms) and “population affected by the disease” (patients who have undergone at least two abortions) following ESHRE guidelines definition 2017^2^. 28 patients were excluded from the study because they lacked most of the analyzes under examination.

### Ethical approval

This study was approved by the Bioethical Committee Board of Tor Vergata University Hospital (number of the Experiments Register: 191/19) and it was carried out in accordance with the Helsinki Declaration ethical principles for medical research. A written informed consent was obtained from all subjects.

### Dataset

The database was created by entering data from diagnostic work-up analysis carried out in our RPL Unit^7^, aimed to identify proven, probable, and doubtful RPL causes^8^.

The features describing each patient were organized in macro-categories:

i. Patients characteristics: age, body mass index (BMI), ethnicity;

ii. Obstetric history: number of at term and preterm pregnancies, number of abortions, live births, time to pregnancy (TTP), type of abortion, clinical classification of abortions, gestational week in which abortions occurred, outcome of past pregnancies;

iii. Environmental factors: radiation, trauma, surgery, smoking;

iv. Infections: Cytomegalovirus, Escherichia Coli, Staphylococci, Mycoplasma, Ureaplasma, Chlamydia, Herpes virus, Toxoplasma;

v. Anatomical causes: congenital uterine anomalies, endometriosis, fibroids, polyps, sinechiae, cervical- isthmic incontinence;

vi. Endocrine causes: diabetes, polycystic ovary syndrome (PCOS), thyroid disorders, prolactin, lutein insufficiency, thyro-stimulating hormone (TSH);

vii. Thrombophilic and immunological causes: Congenital resistance to activated protein C (APCR), protein C, protein S, antithrombin III (AT III), homocysteinemia, lupus anticoagulant (LAC), anti-cardiolipin antibodies (ACL), antinuclear antibodies (ANA), anti-mitochondria antibodies (AMA), extractable anti-nucleus antibodies (ENA), anti-smooth muscle antibodies (ASMA), anti-double strand DNA antibodies, mutation of factor V Leiden, mutation of factor II, mutation in methenyl tetrahydrofolate reductase MTHFR C677T and MTHFR A 1298C, plasminogen activator inhibitor (PAI 1), anti- beta2 glycoprotein 1 antibodies, anti- prothrombin antibodies, anti-annexin V antibodies, anti-gliadin antibodies, anti-transglutaminase antibodies, anti-endomysium antibodies, anti-thyroid peroxidase antibodies (anti-TPO), anti-thyroglobulin antibodies (anti-TG), transaminases (GOT and GPT);

viii. Genetic causes: maternal karyotype, paternal karyotype;

ix. Result of the diagnostic work up.

The analyzes for infectious diseases were defined as:

a) 0 if the cervical-vaginal swabs were negative;

b) 1 if the infection under analysis was in progress;

c) 2 to indicate that the patient has been in contact with the organism in the past or asymptomatic infection. The type of neutralizing antibody was also specified in some infectious tests.

The risk factor associated with the level of smoking was classified into three classes:

1. 0 the patient is not a smoker;

2. 1 the patient smokes a maximum of 10 cigarettes a day;

3. 2 the patient smokes more than 10 cigarettes a day.

As regards the thrombophilic investigations they were presented as a numerical result or as levels of the risk factor.

Immunological investigations were shown as values representing the level of risk or as:

i. negative = 0;

ii. homozygous = 1;

iii. heterozygous = 2

The analyzes of the genetic karyotype were presented as N = negative if no mutation was found, otherwise the type of mutation present was written.

The buffer column and the diagnostic work-up presented descriptive information.

Voluntary termination of pregnancy has been inserted as categories, since it could represent a risk factor because of pro-inflammatory situations caused by the procedure which can alter the site of the embryo implant.

Data regarding infections from Chlamydia, Streptococci, Gardnerella and Candida were using due to their relevant frequency. The features were always discretized from the less relevant to the highest one.

### Data grouping

We grouped multiple features in single features. To ensure the correct contribution of the individual tests to the algorithm, and above all to obtain the lowest number of patients with Not a Number (NaN), in the above-mentioned categories some data have been grouped as a single feature.

For all the items obtained from the swabs concerning streptococci no difference was made between the different species of bacteria.

The triad of aPL antibodies (LAC, ACL and anti-β2 glycoprotein1) was pooled into a single column. In the cases in which the three antibodies were present together (aij = 3) the patient was considered to be affected by APs falling into the “direct cause” classification^9^. If, instead, only one or two of the previous antibodies mentioned were found, the classification was part of the “risk factors” (aij = 1 and aij = 2)^10–13^.

The information of ENA, ASMA, AMA, anti-DNA antibodies, having been evaluated less important than ANA^14–17^, has been grouped into a single datum called “systemic self-immunity” because their presence offers an evaluation of the autoimmune system’s activation capabilities. The data of the feature may appear as 1,2,3,4 indicating the number of types of antibodies present. This grouped feature was identified as a risk factor of equal value to other exams taken as single test, such as the ANA.

The two MTHFR and PAI-1 mutations have been grouped into a single datum, which is a sum of the three data mentioned above: excluding these data from the study would have been a bias, since, even if they don’ t have the same weight as Factor II or Factor V mutations in the coagulation field, they could be considered relevant if their aberrations occur together in a single patient^18–22^.

Anti-gliadin, anti-transglutaminase, anti-endomysium antibodies have been grouped together as a single category called “suspected celiac disease”, with the sum of the aforementioned antibodies present, because of their debated potential role in RPL^23,24^.

Anti-TPO and anti-TG antibodies were grouped in a single datum called “autoimmune thyroiditis”, because of their controversial role as an independent risk factor for RPL, beyond hypothyroidism clinical manifestation^25–28^.

In these last two data, a “non-zero” represents the presence of a risk factor for the patient.

The mutations of the maternal and paternal karyotype were grouped in a single voice called “parental karyotype”.

### Discretization of continuous features

The continuous features (age, BMI, APCR, protein C, protein S, AT III, homocysteinemia, TSH) presented a high presence of NaN because the examination was not prescribed, or because it was evaluated within the normal range and therefore not inserted.

We discretize the data and assume the hypothesis that every NaN corresponded to a value of that feature in the normal range and therefore to the lowest risk class, since no clinical indications were found for the test to be performed in those particular patients.

For all the features, risk classes have been created around the normal value with (class 0), up to the major percentage difference (class 4). Differently, for the BMI the conventional classes have been followed: class 0 normal weight, class 1 underweight, class 2 extreme thinness, class 3 overweight, class 4 obesity. The extreme thinness is considered a lower risk factor compared to the overweight.

For all the other continuous features, risk classes have been created around the normal value with (class 0), up to the major percentage difference (class 4).

Table 9 shows the discretization performed and the number of NaN present in each feature.

**Table 9:** Discretization of continuous features.

Risk Classes	0	1	2	3	4	NaN for each feature
**Age (years)**	18–27	28–32	33–37	38–42	>42	14
**APCR (%)**	2.4–3.5	1.84–2.39 & 3.51–4.05	1.28–1.83 & 4.06–4.61	0.16–1.27 & 4.62–5.73	<0.16 & ≥ 5.74	393
**Protein C (%)**	70–130	55–69.99 & 130.01–144.99	40–54.99 & 145–159.99	25–39.99 & 160–175	<25 & ≥ 175	271
**Protein S (%)**	53–109	38–52.99 & 109.01–123.99	23–37.99 & 124–138.99	8–22.99 & 139–154	<8 & ≥ 154	263
**AT III (%)**	80–120	65–79.99 & 120.01–134.99	50–64.99 & 135–149.99	35–49.99 & 150–165	<35 & ≥ 165	364
**Omocysteinemia (µM)**	5–12	4–4.99 & 12.01–14.99	3–3.99 & 15–17.99	2–2.99 & 18–21	<2 & ≥ 21	272
**TSH (mU/ml)**	0.5–3.8	0.4–0.49 & 3.81–4.79	0.3–0.39 & 4.8–7.79	0.1–0.29 & 7.8–17.99	<0.1 & ≥ 18	200
**BMI (Kg/m**^**2**^**)**	18.5–24.99	16.5–18.49	<16.5	25–30	>30	143

Continuous features were discretized in 5 risk classes ranging from normal (risk class 0) up to high risk (risk class 4). The last column shows the number of NaN (Not a Number) for each feature present in the dataset. The NaN were set to 0 because they corresponded to the case in which the doctor found it was not necessary that exam for that patient and so the feature was in the normal range (see Materials and Methods, Discretization of continuous features).

### Description of our diagnostic work-up features vs diagnostic screening features recommended by ESHRE guidelines

As a further step, through the algorithm, we have compared the procedure recommended by the most recent guidelines in this field (ESHRE guidelines 2017), based on exams that have reached an evidence-based level versus the extended diagnostic work-up carried out in our RPL outpatient clinic (Table 7).

### Diagnostic work-up recommended by the guidelines

As mentioned in the previous paragraph, the decision to perform clinical investigations depends on the patient’s medical history. The diagnostic tests must be performed only if the result of this examination offers a more in-depth view of the prognosis or if the established cause can be treated efficiently. The rising costs of health care are due to ineffective medical tests. In clinical practice too many diagnostic tests and unproven treatments are performed.

According to the guidelines, the diagnostic work-up should include:

- Investigation of the medical history of patients;

- Investigation of patients’ life habits;

- Genetic analysis of maternal and paternal karyotype. In recent years, new techniques are being developed, including the use of FISH probes and above all the Array-CGH, able to assess the presence of any chromosomal anomalies at the entire genome level in a single experiment. The disadvantage of the latter lies in the cost, much greater than the high resolution karyotype;

- Anatomical evaluation of patients with a combination of hysteroscopy and laparoscopy assessment, if needed, that assess the internal and external profiles respectively. From their comparison we can arrive at a correct diagnosis and for this reason their combination is considered the gold standard. However, being invasive tests, it is worthy to make the diagnosis with a three-dimensional ultrasound.

- Endocrinological evaluation for the following dysfunctions: polycystic ovary syndrome (following the diagnostic Rotterdam criteria); diabetes, diagnosed through the oral glucose tolerance test (OGTT) or the glycated hemoglobin test; dysthyroidisms evaluation through checking the level and production of TSH; antiphospholipid antibody syndrome by evaluating the presence of LAC, ACL and anti-β2 glycoprotein in serum or blood on two or more different evaluation^2^.

### Diagnostic work-up performed at our RPL Unit

The diagnostic work-up performed at the Tor Vergata University Hospital is more detailed than the one recommended by the guidelines.

In addition to the procedure introduced in the previous paragraph, the following tests are also performed:

- Evaluations about uterine infections via vaginal and / or cervical swabs;

- Evaluation of thrombophilias and immunological causes such as: mutation of factor V Leiden; Factor II mutation; mutations in MTHFR and PAI-1; anti-annexin V antibodies; APCR, Protein C, Protein S, AT III, homocysteinemia, evaluation of celiac disease; ANA and Systemic autoimmunity through ASMA, ENA, anti-DNA antibodies, AMA; evaluation of thyroid antibodies.

All these analyses are not recommended, but they could explain, not each taken alone, but in a threshold model approach, uRPL cases, considering uRPL as a multifactorial disease.

Furthermore, as previously stated, not all the examinations performed seem to represent a direct cause of RPL, but could be involved in pro-inflammatory actions which, in the early stages of pregnancy, can lead to an unfavorable outcome of gestation.

### Data analysis

For the data analysis, we used a supervised learning algorithm known as Support Vector Machines^29^. We analyzed two different problems. The first was a two-class problem were a class is represented by patients with 0–1 abortions and the other class is represented by patients with > =2 abortions. We further investigated the problem of studying a four-class problem to achieve a risk stratification. The first class was represented by patients with 0 abortion, the second by patients with 1 abortion, the third by patients with 2–3 abortions and the fourth by patients with 4 or more abortions. In both cases, we found a very unbalanced dataset between the classes. Hence, we needed to perform a bootstrap to generate samples of the minority classes and therefore to get a balanced dataset. To carry out this bootstrap operation we used the MATLAB function ADASYN^30^.

This function works only in a two-class context. The bootstrap was made in the four-class problem taking as reference the most represented class (the third class).

### Feature Selection

To select the best feature set, we adopted the same approach for the two-class and four class problem. In particular, we included in the subset the features that showed an AUC value higher than 0.55. It was interesting to note that the same features are selected for both the task (Table 10).

**Table 10:** The first and the second column contain the number and the name of the features. The third column reports which features are suggest from the guidelines. The third column reports which features are taken in our work-up. The last column reports which feature are selected by the feature selection algorithm.

FEATURES		Work-up Guidelines	Our Work-up	Features selected
1	Age	✓	✓	✓
2	BMI	✓	✓	✓
3	Smoke	✓	✓	✓
4	Voluntary termination of pregnancy	✗	✓	✓
5	Cytomegalovirus infection	✗	✓	✓
6	Escherichia Coli infection	✗	✓	✗
7	Staphylococcus infection	✗	✓	✗
8	Mycoplasma infection	✗	✓	✗
9	Ureaplasma infection	✗	✓	✗
10	Herpes virus infection	✗	✓	✗
11	Toxoplasma infection	✗	✓	✓
12	Steptococcus infection	✗	✓	✗
13	Gardnerella infection	✗	✓	✗
14	Candida infection	✗	✓	✗
15	Bicorporeal uterus-class U3	✓	✓	✗
16	Bicorporeal uterus-classe U3bC2	✓	✓	✗
17	Septate uterus-class U4	✓	✓	✗
18	Hemi uterus-class U4	✓	✓	✗
19	Endometriosis	✗	✓	✗
20	Uterine Fibroids	✓	✓	✓
21	Intrauterine Synechiae	✓	✓	✗
22	Cervical-isthmic incontinence	✓	✓	✗
23	Endometrial Polyps	✓	✓	✗
24	Diabetes	✗	✓	✓
25	PCOS (Polycystic Ovary Syndrome)	✗	✓	✗
26	Thyroid disorders	✓	✓	✓
27	APCR (Activated Protein C Resistence)	✗	✓	✓
28	Protein C	✗	✓	✓
29	Protein S	✗	✓	✓
30	Antithrombin III	✗	✓	✗
31	Antiphospholipid antibodies:	✓	✓	✓
	- LAC (Lupus Anti Coagulant)	✓		
	- ACL (anti-cardiolipin Antibodies)	✓		
	- Ab anti-β2-GPI (anti-β2glycoprotein 1 Antibodies)	✗		
32	ANA (Anti-nuclear antibodies)	✗	✓	✓
33	Systemic autoimmunity: - ENA (Extractable Nuclear Antigens) - ASMA (Anti-Smooth Muscle Antibodies) - AMA (Anti-mitochondrial Antibodies) - Ab anti-dsDNA (Anti-double stranded DNA Antibodies)	✗	✓	✓
34	Factor V Leiden Mutation	✗	✓	✗
35	Factor II Mutation	✗	✓	✗
36	Trombophilic polymorphisms: - MTHFR (Metilen Tetra-HydroFolate Reductase) C677T - MTHFR A1298C - PAI 1 (Plasminogen Activator Inhibitor 1)	✗	✓	✓
37	Homocysteine	✗	✓	✓
38	Anti-annexin V Antibodies	✗	✓	✗
39	Suspected celiac disease: - Anti-gliadin Antibodies - Anti-endomysium Antibodies - Anti-transglutaminase Antibodies	✗	✓	✗
40	Thyroid autoimmunity:	✓	✓	✓
	- Ab anti-TPO (Anti-Thyroid Peroxidase Antibodies)	✓		
	- Ab anti-TG (Anti-Thyroglobulin Antibodies)	✗		
41	Maternal chromosomal abnormalities	✗	✓	✗
42	Paternal chromosomal abnormalities	✗	✓	✗
43	TSH (Thyroid-Stimulating Hormone)	✓	✓	✗

The outcome of SVM model, the number of preceding pregnancy losses, is considered as a fundamental feature for the prognosis by the guidelines.

### SVM training

We used the same cross-validation strategy for both two-classes and four-classes problems.

The performances of SVM classification model has been evaluated using a k-fold cross-validation procedure. In particular, the original dataset was divided randomly into 11 random blocks of 200 samples. At every step, one block was used for test and the other data were used for training the model. We iterated this procedure a thousand times reporting as results mean and standard deviation of each class.

In order to solve the four-class problem with an SVM classification model, we adopted the one-vs-one strategy, hence we trained 6 different two-class models. The test sample will be assigned to the class characterized by the highest probability (lowest aggregation loss of all the six classifiers).

All the features were autoscaled using the mean and the standard deviation of the training set. We used a radial basis function as Kernel Function. All the other parameters were set to default (fitcecoc MATLAB function).

### Data output

In order to guarantee a fluent use of the algorithm by the healthcare providers, an interface has been created, in which the results are inserted as a line vector with as many components as the total number of exams that can be performed.

## Data Availability

The dataset analyzed during the current study is available from the corresponding author on reasonable request
